# Outcome of Matrix Rotation Versus Single Incision Lateral Sulcus Mammoplasty in Upper Quadrant Breast Carcinomas

**DOI:** 10.3390/medicina61091609

**Published:** 2025-09-05

**Authors:** Emad M. Abdelrahman, Sherif M. Mohsen, Amr G. Mohamed, Mostafa S. Abdeen, Mohamed A. Elsayed, Zizi M. Ibrahim, Osama R. Abdelraouf, Hassan Hegazy, Mahmoud G. Abdelhalim

**Affiliations:** 1 Department of General Surgery, Faculty of Medicine, Benha University, Benha 13518, Egypt; amr.g.m.2010@gmail.com (A.G.M.); mahmoud.gouda@fmed.bu.edu.eg (M.G.A.); 2 Department of General Surgery, Faculty of Medicine, Ain Shams University, Cairo 11566, Egypt; dr.sheriefmohsen@outlook.com; 3 Plastic Surgery Unit, Department of General Surgery, Faculty of Medicine, Benha University, Benha 13518, Egypt; mustafa.abdeen@fmed.bu.edu.eg (M.S.A.); mohamed.elsayed@fmed.bu.edu.eg (M.A.E.); 4 Department of Rehabilitation Sciences, College of Health and Rehabilitation Sciences, Princess Nourah bint Abdulrahman University, P.O. Box 84428, Riyadh 11671, Saudi Arabia; zmibrahim@pnu.edu.sa; 5 Physical Therapy Program, Batterjee Medical College, Jeddah 21442, Saudi Arabia; pt4.jed@bmc.edu.sa; 6 Department of Otolaryngology, Faculty of Medicine, Tanta University, Tanta 31527, Egypt; hassan.hegazy@ksiu.edu.eg

**Keywords:** MRF, SLIM, oncoplasty, breast cancer

## Abstract

*Background and Objectives:* The term “oncoplastic breast surgery” (OBS) incorporates plastic and oncologic concepts. Through the application of diverse mammoplasty approaches, the remaining breast tissue can be reconstructed, thereby enabling more extensive resections to be achieved with oncologically safe, margin-free outcomes. This study aims to assess the efficacy of the single incision lateral mammoplasty (SILM) technique as an oncoplastic approach for managing breast cancer located in the outer quadrant, in comparison with the matrix rotation flap (MRF) technique. *Materials and Methods*: This prospective randomized controlled study comprised 68 patients, who were randomized into two groups scheduled to undergo breast surgery: Group A constitutes the matrix rotation flap MRF group and Group B represents the single incision lateral sulcus mammoplasty (SLIM) group. A follow-up was planned for postoperative complications and esthetic outcomes. *Results*: The mean age of patients in Group A was 51.4 ± 9.4 years, compared with 52.6 ± 8.1 years in Group B. A total of 14.7% and 11.8% of patients in Group A reported a hematoma or seroma, respectively, which were higher than what was reported in Group B, where a hematoma and seroma were reported in 5.9% of patients. Additionally, 32.4% and 50% of patients in Groups A and B, respectively, reported excellent satisfaction. The evaluation with the Vancouver Scar Scale (VSS) revealed that esthetic outcomes were significantly better in Group B. *Conclusions*: Compared to the MRF procedure, the SLIM results in a much lower rate of postoperative hematoma, minor seroma, minimum blood loss, reduced areolar deviation, and improved breast symmetry. Both the MRF and SLIM techniques yield acceptable cosmetic outcomes. However, a longer-term follow-up is necessary to establish the definitive oncological equivalence between techniques.

## 1. Introduction

With a cure rate of over 95% in the early stages of breast cancer, multidisciplinary team therapy (MDT) significantly improves outcomes and prognoses [1].

Breast-conserving surgery (BCS), consisting of wide local tumor excision followed by adjuvant radiotherapy, offers comparable survival outcomes to mastectomies and is considered a safe and effective alternative [2,3].

Neoadjuvant chemotherapy (NACT) facilitates tumor downstaging in cases where the tumor-to-breast ratio initially precludes breast-conserving surgery (BCS). In addition, NACT may reduce the extent of the axillary surgery required in patients with node-positive disease [4,5].

Approximately 30–40% of patients undergoing breast-conserving surgery (BCS) followed by adjuvant radiotherapy experience breast deformity and suboptimal cosmetic results due to the surgical cavity and radiation effects [6].

Oncoplastic breast surgery, developed in 1996, combines principles of plastic surgery with breast-conserving surgery (BCS), thereby facilitating more extensive resections without compromising esthetic or oncological results [7,8]. Additionally, from a physical, emotional, and psychological perspective, undergoing these treatments enhances the quality of life for women [9].

Sixty percent of breast cancers occur in the lateral breast quadrants, and many oncoplastic techniques have been developed for these cancers. Numerous methods have already been proven to combine oncological safety with acceptable esthetic outcomes, such as round block, inferior pedicle reduction, Matrix rotation, racquet mammoplasty, and single incision lateral sulcus mammoplasty. The selection of the surgical technique is influenced by multiple factors, including the tumor size, the breast volume, the anticipated cosmetic outcome, and the surgeon’s level of expertise. Most of these techniques cause scarring near the nipple–areola complex (NAC), leading to its deviation, which can significantly diminish esthetic results and complicate the management of upper outer quadrant breast masses [10,11,12].

When larger tumors are removed, the nipple–areolar complex (NAC) often deforms and shifts laterally and upward. This deformity is associated with increased patient dissatisfaction and can sometimes be worsened by postoperative radiation therapy [13,14].

This study aims to evaluate and compare the single incision lateral mammoplasty (SILM) technique as an oncoplastic procedure for managing breast carcinoma in the lateral breast quadrant with the matrix rotation flap (MRF) technique. The comparison focuses on cosmetic and oncologic outcomes and patient satisfaction.

## 2. Materials and Methods

### 2.1. Study Design

This study was carried out between April 2021 and June 2024 in accordance with the ethical standards outlined in the Declaration of Helsinki. Sixty-eight female patients with breast cancer in the outer quadrants of the breast who were eligible for wide local excision and reconstruction using either MRF or SLIM were included.

Exclusion criteria included individuals with advanced or metastatic breast cancer. Patients with a history of prior irradiation or contraindications to radiotherapy, as well as those with scleroderma or collagen diseases, were excluded. Additionally, patients who declined to participate in the study were also excluded.

Randomization was conducted with Random Allocation Software (version 1.0, 2011), assigning patients equally to the two study groups. The software generated an unpredictable random sequence and implemented it in a way that conceals the treatments until patients are officially assigned to their respective groups.

An independent investigator performed the randomization. Patients, surgeons, outcome assessors, and data analysts were blinded to group allocation. This single-blind randomized controlled trial was performed after participants received detailed information on both surgical techniques and their anticipated outcomes. Clinical trial registry at https://clinicaltrials.gov/study/NCT07022509 (accessed on 18 June 2025).

**Figure 1:**
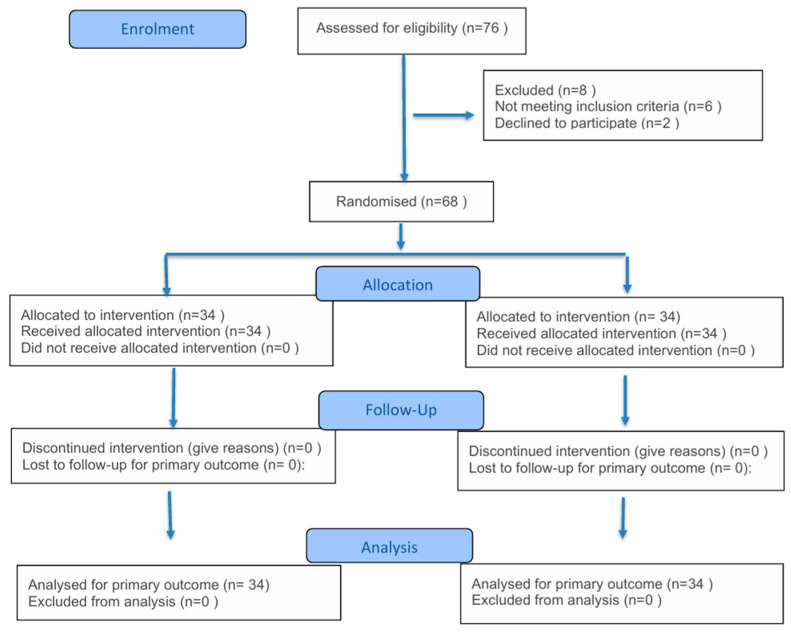
Consort flow chart.

Group A (*n* = 34) underwent matrix rotation flap MRF technique, and Group B (*n* = 34) underwent single incision lateral mammoplasty (SILM) technique [Figure 1].

This multicenter study was carried out at the Departments of General Surgery at Benha University and Ain Shams University. The study design was reviewed and approved by the institutional ethics committee, and written informed consent was obtained from all participants. Preoperative assessment included detailed history taking, comprehensive breast and axillary examination, and bilateral sonomammography for all patients.

The malignant nature of the lesion was verified by a true-cut needle or excisional biopsy. Patients with locally advanced T4 tumors, inflammatory carcinoma, or distant metastases were not eligible for inclusion. Individuals with multicentric breast cancer in more than one quadrant and diffuse microcalcifications were also excluded.

### 2.2. Surgical Procedure

#### 2.2.1. Preoperative Considerations and Flap Design

To ensure an accurate assessment of the anatomical landmarks, the markings were made while the patient was upright, either sitting or standing. Breast defect volume was assessed by marking the lateral breast border and resection margins. The flap was then outlined for transposition, with a length of 8–11 cm measured from the deformity edge to the posterior axillary line.

The flap width, typically ranging from 3 to 6 cm, was determined to ensure tension-free donor site closure. Flap thickness—generally ranging from 2 to 5 cm, depending on the amount of available skin and subcutaneous tissue—was estimated using the pinching test. The base of the flap, located at the anterior axillary fold, is usually smaller than its sides, which are more convex to maximize tissue harvest. Perforators cannot be detected with handheld Doppler ultrasonography.

The patient was positioned supine, with the ipsilateral arm abducted at 90° to facilitate lymph node surgery (dissection and/or sentinel lymph node biopsy). Preoperative planning included assessment of breast size, tumor size, tumor location, and estimated defect volume, followed by repeated confirmation of surgical markings.

##### Group A: MRF Group (Figure 2 and Figure 3)

**Figure 3:**
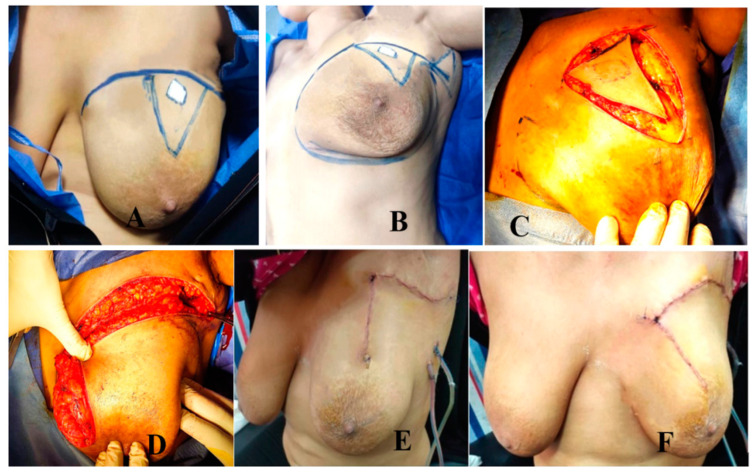
MRF (**A**,**B**) marking of the tumor and the planned incision. (**C**) Excision of the tumor down to the pectoral fascia. (**D**) Mobilization of the flap into the defect. (**E**,**F**) Esthetic outcome.

**Operative technique (Figure 3)**

Following the induction of muscle relaxation and general endotracheal anesthesia. The operation was performed in three stages as follows:

**The first stage is tumor resection:** When a tumor is removed using the typical quadrantectomy procedure, the entire tumor and the surrounding safety margin are dissected. The tumor was excised down to the pectoral fascia, with a minimum safety margin of 1 cm maintained in all directions. Clips are used to mark the tumor bed. The specimen is submitted for frozen section analysis to assess the radial margins after their boundaries are indicated with threads. A wider re-excision is performed if infiltration of the specific margin is present. In cases where the tumor extended near the skin surface, excision included the lesion and the corresponding overlying skin.

**The second stage involves axillary surgery:** As outer quadrant lesions are anatomically adjacent to the axilla, surgery was performed through the same incision used for tumor resection. To access the clavico-pectoral fascia, which was exposed and opened to reach the axillary area, the incision is deepened downward. Depending on each patient’s preoperative decision, either axillary dissection or sentinel lymph node biopsy was performed. Axillary dissection was performed in cases where sentinel lymph node biopsy yielded positive results. Particular attention was paid to preserving the thoracodorsal pedicle to maintain the option of future latissimus dorsi (LD) flap reconstruction. When axillary dissection was undertaken, a single drain was placed in the axilla.

**The third stage involves flap harvesting and reconstruction:** The flap length was measured to ensure that its tip could adequately reach the most distant margin of the defect. Flap width was estimated using the pinching test, which allowed for the determination of the required tissue volume while ensuring tension-free donor site closure. To maintain sufficient vascularity via the dermal and subdermal plexus, the flap base was designed with a minimum width of 3 cm.

Flap harvesting was performed by incising the skin and subcutaneous tissue and beveling the surrounding fat to maximize tissue volume. The fascia overlying the latissimus dorsi and serratus anterior muscles was elevated together with the flap. To achieve sufficient flap mobilization into the defect, selected lateral intercostal perforators were ligated and divided.

The skin covering the excised flap is de-epithelialized to ensure its vascularity. In cases where the overlying skin was excised with the tumor, the remaining flap was de-epithelialized. The tissue bridge between the donor site and the defect was divided, and a skin paddle was designed and tailored to conform to the dimensions of the defect.

Following rotation and insertion of the flap into the breast defect, the flap margins were secured to the pectoral fascia using 2/0 Vicryl sutures. Usually, only one surgical drain is left in the breast area, not at the flap donor site. The incision made at the donor location is closed in layers.

##### Group B: Lateral Mammoplasty Group (Figure 4 and Figure 5)

**Preoperative planning and marking**

A safety margin of 1 cm was used to localize the tumor. An imaginary line was then drawn from the inframammary fold upward toward the outer border of the pectoralis major muscle, delineating the lateral boundary of the breast. On this line, a 7–10 cm incision was deeply marked facing the tumor’s location. The procedure was performed in three phases.

**The first stage was tumor excision.** After opening, the incision was deepened until it reached the glandular tissue. Following the creation of the upper breast skin flap, the entire tumor and the surrounding safety margin were dissected. The tumor was excised in a wedge-shaped fashion down to the pectoral fascia, with the apex directed toward the lateral breast margin and the base oriented toward the areola, ensuring a minimum safety margin of 1 cm circumferentially. The tumor bed was subsequently marked with clips. Threads were used to mark the specimen’s edges before it was frozen and sent for histological analysis to assess the margin. Wider re-excision was performed in cases of specific margin infiltration.

**Figure 4:**
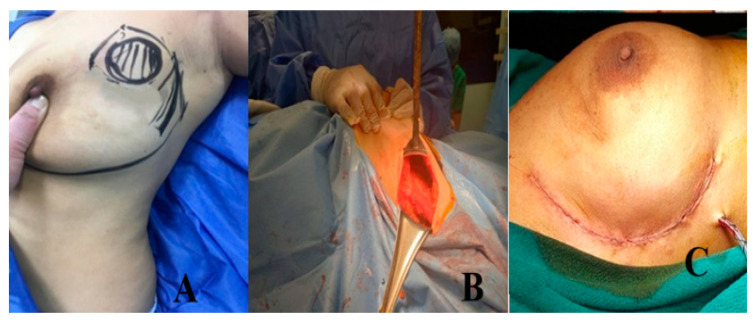
SLIM Technique: (**A**) marking the mass and incision; (**B**) excision of the mass down to the pectoral fascia; and (**C**) closure of the wound over the drain.

**The second stage was axillary surgery.** The clavi-pectoral fascia, which was exposed and opened to access the axillary space, was reached by deepening the bottom edge of the incision all the way down, Figure 4. Depending on the preoperative planning, axillary management consisted of either a sentinel lymph node biopsy or an axillary dissection. In cases with a positive sentinel lymph node biopsy, axillary dissection was subsequently performed.

**Closure is the third stage.** At a distance of 4 cm, the upper and lower dermoglandular flaps that encircle the tumor bed are released upward from the skin flaps above and downward from the underlying pectoral fascia. Then, 2/0 polyglycolic acid is used to mimic the dermoglandular flaps in two layers. As usual, the skin and subcutaneous tissue are sealed off. Unless axillary clearance was completed, drains were not used.

### 2.3. Follow-Up and Outcomes

All patients were discharged on the first postoperative day with drains in situ. Drains were removed once the output decreased to less than 50 cc over 24 h. To plan adjuvant treatment and assess surgical complications, patients were evaluated in the outpatient clinic at one and two weeks.

All patients were scheduled for review by our interdisciplinary team every four months during the first two years. Each year, a bilateral sonomammography was performed.

All patients were followed for at least 14 months and up to 36 months for the first included cases. Both postoperative complications and esthetic outcomes were reported.

**The primary outcome** was the removal of UOQ breast tumors with oncological safety and reconstruction using the SILM or matrix rotation technique with minimal complications.

**The secondary outcome** was obtaining acceptable esthetic results with patient satisfaction as the primary focus.

For each patient, operative time, resection margin status, and postoperative complications, including wound infection, wound dehiscence, hematoma, seroma, nipple–areola complex (NAC) changes, skin slough or necrosis, and alterations in NAC sensation were evaluated.

Cosmetic outcomes were assessed based on breast symmetry, scarring, and patient satisfaction and graded using a five-point Likert scale, with scores defined as follows: 1 = excellent, 2 = good, 3 = fair, 4 = poor, and 5 = bad [16]. The Likert scale protocol includes several questions for patients about the size, symmetry, pain or hyperesthesia, NAC shape and position, and scar shape. Meanwhile, the esthetic outcome was determined by assessing breast symmetry, position, NAC distortion, as well as scarring, retraction, and final scar appearance. Four main criteria were used to evaluate scars with Vancouver’s scar scale [17]: height, vascularity, pliability, and pigmentation. The total score ranged from 0, representing normal skin, to 13, indicating the worst possible scar. Cosmetic outcomes were evaluated using the Vancouver Scar Scale (VSS) during the postoperative period (3–6 months). Two independent plastic surgery consultants, blinded to the patients’ clinical details and not involved in the surgical procedures, performed the assessments, and the mean of their scores was considered for analysis.

### 2.4. Statistical Analysis

Sample size was calculated using GPower 3.1 software (Düsseldorf, Germany). Each group initially included 34 patients, accounting for a 20% anticipated dropout rate during the follow-up period. Calculations were based on an effect size of 0.9, a statistical power of 95%, and a type I error rate of 0.05.

Data was analyzed using IBM SPSS software version 21.0 (IBM Corp., Chicago, IL, USA). Statistical significance was set at *p* < 0.05 for all comparisons. Categorical variables are presented as counts and percentages, whereas continuous data are expressed as means with standard deviations or medians with interquartile ranges based on normality assessments. To evaluate differences between groups, continuous data were summarized as mean ± standard deviation and compared using Student’s *t*-test. Categorical and binary variables were analyzed by chi-square or Fisher’s exact tests according to expected cell frequencies. For quantitative data violating normality assumptions, the non-parametric Mann–Whitney U test was applied. All tests were two-sided, with *p* values determining statistical significance. Pearson correlation coefficient (r) measured the linear relationship between a person’s quantitative data, specifically VSS, and patient satisfaction. A strong correlation between two variables is indicated by an (r) value between 0.7 and 1.

## 3. Results

This study enrolled a total of 68 patients. The mean age was 51.4 ± 9.4 years in Group A and 52.6 ± 8.1 years in Group B. There were no significant differences between the groups regarding sociodemographic factors or preoperative tumor characteristics (Table 1).

The operative time was significantly shorter in patients who underwent the SLIM compared to those who underwent the MRF procedure (*p* < 0.001). However, no statistically different mean hospital stay was reported between the two groups, Table 2.

The safety margin of the excised tissue was negative in all included patients, Table 2.

Regarding the assessment of postoperative outcomes, 14.7% and 11.8% of patients in Group A reported a hematoma or seroma, respectively, which were higher rates than those in Group B, where a hematoma and seroma were reported in 5.9% of patients. Wound infection and fat necrosis were also more common in Group A compared to Group B. An areola deviation was observed in patients who underwent the MRF procedure. Breast asymmetry was objectively assessed by comparing the breast size and the position of the nipple–areola complex (NAC) relative to the contralateral breast. Asymmetry occurred in 20.6% of patients in Group A and in 23.5% of patients in Group B. One case in each group experienced local recurrence during follow-up, presenting as a small mass despite negative pathological margins, and received the protocol of adjuvant and neoadjuvant chemotherapy Table 3.

**Figure 6:**
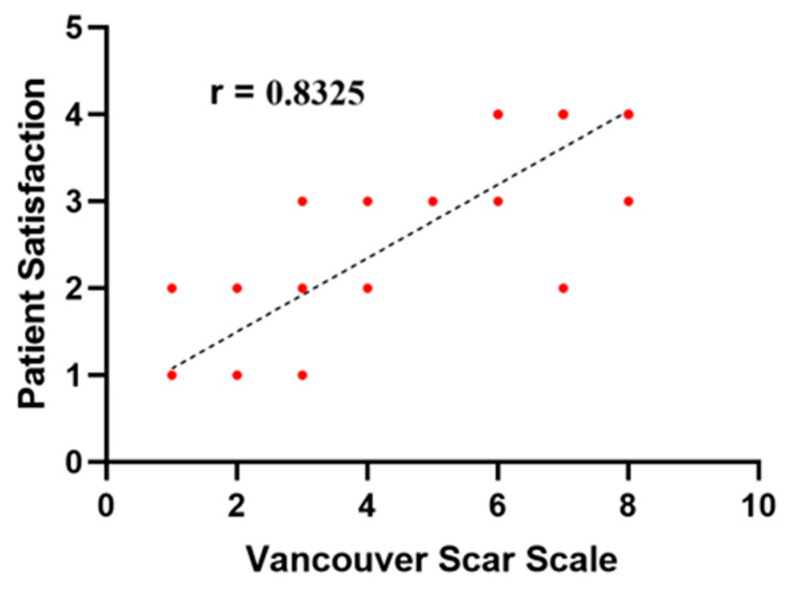
Correlation between VSS and patient satisfaction.

Table 4 summarizes the esthetic outcomes, with excellent satisfaction reported by 32.4% of patients in Group A and 50% in Group B. No cases were reported as having a bad outcome in either group, while only one patient in Group A experienced a poor outcome. The esthetic outcome was significantly better in Group B, as measured by the Vancouver Scar Scale (VSS), compared to Group A. A strong positive correlation was found between patient satisfaction and the VSS (r = 0.832) (Figure 6). The vascularity and pliability of the scar in the VSS were significantly better in the SLIM group.

## 4. Discussion

Breast conservation therapy (BCT), comprising a wide local excision followed by postoperative radiotherapy, offers an effective approach to mitigate the psychological and physical drawbacks associated with mastectomies in breast cancer patients. Additionally, restoring the shape and contour while maintaining oncologic safety is facilitated by integrating plastic surgical techniques into breast cancer surgery [18].

In the present study, 64 female patients were included, with a mean age of 51.4 ± 9.4 years in Group A and 52.6 ± 8.1 years in Group B. The main T stage of the patients was T2, consistent with the results of Lin et al. [19] and many other authors [20,21].

Optimizing healing, functional outcomes, and the overall quality of life in breast cancer patients necessitates a comprehensive, multidisciplinary approach to postoperative care. An adequate recovery depends on the coordinated efforts of a team that includes oncologic surgeons, plastic surgeons, radiation oncologists, physiotherapists, psychiatrists, dietitians, and specialized nursing staff [22,23]. Each discipline plays an essential role in ensuring optimal healing, managing complications, and supporting the patient’s overall wellbeing. Among these, physical therapy is vital in preventing lymphedema, restoring upper limb function, and reducing musculoskeletal pain caused by adjuvant therapies and surgery. Starting a targeted physical therapy program early has been shown to significantly improve mobility, reduce post-mastectomy complications, and enhance long-term patient wellbeing [24,25,26].

An additional popular technique among breast surgeons is the matrix rotation approach. This method is straightforward, provides enough glandular tissue for breast repair and reconstruction, and is most effective for UIQ cancers located away from the NAC. The main drawback of this approach is that the skin in front of the tumor must be removed, even if it is oncologically clear. As a result, the visible breast line crossing the UIQ becomes an unsightly scar [15,19,27].

When it comes to scar concealment, the lateral sulcus incision is among the best options. For laterally positioned early breast cancer, the lateral mammary sulcus approach is a level 1 OPBS in which the lump is removed in two planes, if necessary, after an adipofascial flap or glandular rotational flap is created to seal the defect properly. In a concealed site that is not visible when the patient is seated, the lateral method allows for the tumor removal with an adequate margin of safety. It enables an axillary operation using a single incision [28,29].

In the series by Van Paridon et al. [30], evaluating the outcomes of oncoplastic breast surgery, it was found that postoperative complications included seroma (5.3%), hematoma (4.1%), and breast asymmetry that required revisional surgery in 5.3% of cases. The rate of these complications was lower than that of the present study because their study subjects included both benign and malignant diseases. Additionally, the small sample size of the current study may contribute to the comparatively higher incidence of complications.

According to Al Kelany et al. [28], the results of the SLIM showed that infection was reported in 3.9% of cases, and seroma was reported in 23.4% of cases, which was higher than the findings of the current study. This can be explained by the fact that the included patients’ BMIs were higher than those in the study, aligning with the American Society of Breast Surgeons’ findings, which show a stronger correlation between SSI, seroma, and obesity—a risk factor for SSI following breast surgery. As a result, 15 individuals in Singh et al.’s study [31] were diagnosed with SSIs. Delays in wound healing were observed in four people. Two individuals experienced marginal skin necrosis. During follow-up, four people developed fat necrosis.

Additionally, Fayed et al. [29] found that an SSI was the most common complication (12 cases), and it can be treated medically without surgery.

According to Fayed et al. [29], the lateral sulcus oncoplastic procedure offers a treatment option for patients with breast cancer that is both cosmetically and oncologically acceptable. Although our patients’ follow-up is not enough to fully assess the treatment’s oncologic safety, they were generally satisfied with the cosmetic results, and there was no local recurrence during this period.

Patients in the MRF group (A) experienced hematomas in 14.7% of cases, compared to 5.9% in Group B. This difference was statistically significant (*p* = 0.042). The hematomas resolved with conservative treatment, consistent with Lin et al. [19], who reported that no hematomas were observed during the follow-up period after breast-conserving surgery using matrix rotation flaps.

Based on the findings of this study, Kaviani et al. [32] discovered that when comparing oncoplastic breast surgery (OBS) with breast-conserving surgery (BCS), OBS shows more promise for the future because BCS may not deliver satisfactory cosmetic results for both the surgeon and the patient. In BCS, the cosmetic failure rate is about 30 to 35%, while in OBS, it ranges from 0 to 16%.

Furthermore, as suggested by Massy et al. [33], there is abundant evidence supporting the preservation of the breast contour and shape, with positive esthetic results and patient satisfaction, when using matrix rotating flaps for breast conservation.

In a total of 21.44% of patients who underwent matrix rotation, results were favorable: 50% experienced good results, 21.44% had fair outcomes, and 7.14% encountered negative results, according to Sakr et al. [34], who compared the MRF procedure with Modified Inferior Based Reduction Mammoplasty and reported better esthetic outcomes in patients who had the therapeutic mammoplasty. The findings of this study align with the excellent results seen in 32.4% of cases, which is comparable to the 35% reported by Sakr et al. [34], who used the matrix rotation technique for UIQ lesions. This may be explained by the method’s focus on UOQ lesions, which required more lateral scarring and less dissection.

The second problem is that the matrix rotation technique produces less-than-ideal cosmetic results for large ptotic breasts. Therefore, it is considered suitable for mild to moderate ptosis cases [35].

Letzkus et al. [27] reported cosmetic outcomes of the Matrix Rotational Advancement flap, with 28.6% of patients rated as excellent, 51.8% as good, 16.1% as fair, and 3.5% as poor.

The correlation between the observer’s evaluation and the patient’s satisfaction and assessment is essential for assessing the esthetic outcome as well as the validity and reliability of any evaluation scores. The VSS is one of the most reliable and widely used assessment tools for postoperative outcomes. In this study, a strong correlation was observed between the VSS and patient satisfaction, indicating the reliability of the results [36].

A systematic review was conducted of 25 papers reporting cosmetic outcomes in 1962 patients who underwent oncoplastic breast-conserving surgery. Excellent, good, fair, and poor results were observed in 55.3%, 32.0%, 9.5%, and 5.4% of patients, respectively. Most research indicates that over 90% of patients who had OBCS experienced satisfactory cosmetic outcomes [32,37,38]. This finding aligns with the current results, which also showed that most patients had positive outcomes. The matrix rotation technique resulted in only one patient reporting a negative result. Patients who underwent the SLIM had significantly better esthetic results, likely due to the absence of a visible scar over the breast in the SLIM group.

Abdelrahman et al. [39] evaluated esthetic outcomes of volume replacement techniques, including the latissimus dorsi flap and thoracodorsal artery perforator flap, reporting acceptable results for both approaches. These findings are consistent with the outcomes of the present study, despite the greater technical complexity encountered in their study.

In their study in 2021, Cantürk et al. [12] described the optimal planned oncoplastic procedure based on the tumor location, considering the breast size and ptosis as key factors for both esthetic outcomes and oncological safety. They highlighted the inferior pedicled reduction mammoplasty as an important technique for UOQ breast lesions in medium-sized and large ptotic breasts.

No patients in either group in the current study reported needing to reposition or synchronize the NAC, which aligns with the findings of Hamed et al. [40], who discovered that patients who underwent the matrix rotation did not require revisional surgery to improve the esthetic outcome.

## 5. Limitations

The primary limitation of this study was the relatively small number of well-designed prior studies available for direct comparison. Other limitations included subjectively assessed outcomes and the limited follow-up for oncologic endpoints, as some cases were followed for only 14 months. This short follow-up period was a weakness of this study, since oncological safety requires an evaluation over a longer duration. The study population’s characteristics that might impact generalizability (e.g., BMI range) and the inherent variability in a multicenter study also pose limitations. Additionally, differences among centers and surgeons introduce potential variability, and the involvement of multiple centers increases the likelihood of external variability and operator-related bias.

## 6. Conclusions

Compared to matrix rotation, single incision lateral mammoplasty results in a significantly lower rate of postoperative hematoma, minor seroma, minimal blood loss, reduced areolar deviation, and improved breast symmetry. Both MRF and SLIM techniques produce acceptable cosmetic outcomes. However, longer-term follow-up is needed to determine the definitive oncological equivalence between the techniques.

## Figures and Tables

**Figure 2:**
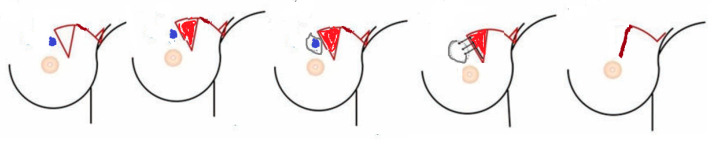
Schematic diagram for MRF [modified from Wijesinghe K et al. [15]. Blue is the mass, red is the triangle to be de-epethelialized and mobilized.

**Figure 5:**
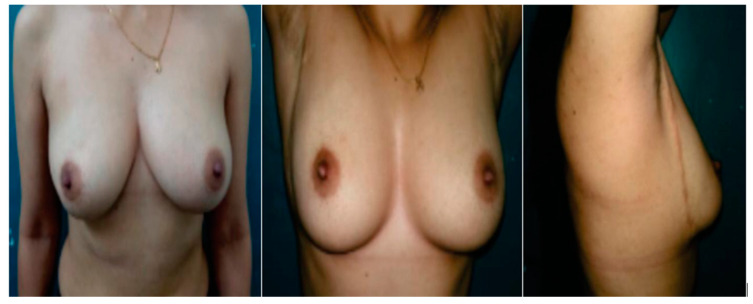
Esthetic outcome following SLIM Technique.

**Table 1:** Patients’ demographic data and tumor characteristics.

Variable		Group A Matrix Rotation *n* = 34	Group B SLIM *n* = 34	*p* Value
**Age**	Mean ± SD	51.4 ± 9.4	52.6 ± 8.1	0.575
**BMI**	Mean ± SD	29.8 ± 3.11	28.3 ± 4.2	0.99
**Comorbidities**				
**Smoking**	*n* (%)	6 (17.7%)	7 (20.6%)	0.9
**Diabetes mellitus**	*n* (%)	4 (11.8%)	3 (8.8%)	0.82
**Hypertension**	*n* (%)	4 (11.8%)	5 (14.7%)	0.85
**Ischemic heart disease**	*n* (%)	2 (5.9%)	2 (5.9%)	1.00
**Tumor Characteristics**				
**Tumor Size**				
**T1**	*n* (%)	7 (20.6%)	5 (14.7%)	0.752
**T2**	*n* (%)	21 (61.7%)	23(67.6%)	0.8
**T3**	*n* (%)	6 (17.7%)	6 (17.7%)	1.00

**Table 2:** Operative findings.

Variable		Group A MRF (*n* = 34)	Group B SLIM (*n* = 34)	*p* Value	Mean Difference (95% CI)
**Operative time (Min)**	Mean ± SD	124.6 ± 23.2	106.3 ± 17.3	<0.001 *	18.3 (8.39–28.21)
**Hospital stay (Days)**	Mean ± SD	2.7 ± 0.89	2.3 ± 0.76	0.051	0.4 (0.0007–0.8)
**Mean postoperative follow-up (Months)**	Mean ± SD	13.2 ± 2.4	13.6 ± 3.1	0.554	0.4 (−1.47–0.94)
**Safety margin**	*n* (%)			1.00	----
**Positive**		0 (0%)	0 (0%)		
**Negative**		34 (100%)	34 (100%)		

* means statistically significant.

**Table 3:** Postoperative sequelae.

Postoperative Complications		Group A MRF (*n* = 34)	Group B SLIM (*n* = 34)	*p* Value	Odds Ratio (95% CI)
**Hematoma**	*n* (%)	5 (14.7%)	2 (5.9%)	<0.001 *	2.759 (0.51–14.5)
**Seroma**	*n* (%)	4 (11.8%)	2 (5.9%)	0.014 *	2.133 (0.46–11.73)
**Wound infection**	*n* (%)	4 (11.8%)	1 (2.95%)	<0.001 *	4.4 (0.65–55.23)
**Marginal skin necrosis**	*n* (%)	1 (2.95%)	1 (2.95%)	1.00	1.0 (0.05–19.52)
**NAC necrosis**	*n* (%)	0 (0%)	0 (0%)	1.00	----
**Impaired NAC sensation**	*n* (%)	0 (0%)	0 (0%)	1.00	----
**Fat necrosis**	*n* (%)	4 (11.8%)	2 (5.9%)	0.014 *	2.133 (0.46–11.73)
**Areola deviation**	*n* (%)	5 (14.7%)	1 (2.95%)	<0.001 *	5.69 (0.68–68.76)
**Asymmetry**	*n* (%)	7 (20.6%)	8 (23.5%)	0.072	0.843 (0.28–2.45)
**Local recurrence**	*n* (%)	1 (2.95%)	1 (2.95%)	1.00	1.0 (0.05–19.52)
**Time of recurrence (months)**	Mean	11	13.		0.083
**Distant metastasis**	*n* (%)	0 (0%)	0 (0%)		----

* means statistically significant.

**Table 4:** Assessment of cosmetic outcomes.

Variable		Group A MRF (*n* = 34)	Group B SLIM (*n* = 34)	*p* Value
**Likert Scale**				
Excellent	*n* (%)	11 (32.4%)	17 (50%)	<0.001 *
Good	*n* (%)	13 (38.2%)	14 (41.2%)	0.9
Fair	*n* (%)	9 (26.5%)	3 (8.8%)	<0.001 *
Poor	*n* (%)	1 (2.95%)	0 (0%)	0.92
Bad	*n* (%)	0 (0%)	0 (0%)	1.00
**Vancouver Scar Scale**	Median (IQR)	3.5 (4)	2.5 (2)	<0.001 *
**Vancouver Scar Components’ Score**				
**Vascularity**	Mean ± SD	1.2 ± 0.34	0.55 ± 0.13	0.01 *
**Pigmentation**	Mean ± SD	1.3 ± 0.26	1.1 ± 0.18	0.86
**Pliability**	Mean ± SD	2.1 ± 0.32	0.7 ± 0.12	0.001 *
**Height (mm)**	Mean ± SD	1.4 ± 0.22	1.1 ± 0.2	0.63

* means statistically significant.

## Data Availability

Data from this work are available from the authors and have not been published elsewhere.
